# Multidisciplinary Surgical Treatment of Hepatic Abscess in a Geriatric Dog with Congenital Extrahepatic Portosystemic Shunt

**DOI:** 10.3390/vetsci13010037

**Published:** 2026-01-01

**Authors:** Kyu-Duk Yeon, Jin-Young Choi, Ji-Hyeok Seo, Joong-Yeon Choi, Chang-Hun Moon, Jung-Hyun Kim

**Affiliations:** 1 Department of Veterinary Internal Medicine, College of Veterinary Medicine, Konkuk University, Seoul 05029, Republic of Korea; gundam223@naver.com; 2 SNC Animal Medical Center, Seoul 06244, Republic of Korea; aditee67@naver.com (J.-Y.C.); nurong3000@hanmail.net (J.-H.S.); choi.joongyeon@sncamc.kr (J.-Y.C.); doorwindow@sncamc.kr (C.-H.M.); 3 KU Animal Cancer Center, Konkuk University Veterinary Medical Teaching Hospital, 120, Neungdong-ro, Gwangjin-gu, Seoul 05029, Republic of Korea

**Keywords:** dog, hepatic abscess, extrahepatic portosystemic shunt, multidrug resistance, cholecystectomy, hepatic lobectomy, geriatric patient

## Abstract

Hepatic abscesses are uncommon in dogs and typically develop secondary to biliary tract disease or ascending bacterial infections. Although congenital extrahepatic portosystemic shunt (EHPSS) is known to impair hepatic perfusion and immune clearance, its potential role in predisposing geriatric dogs to hepatic abscess formation has not been previously reported. This case report describes the diagnostic approach, therapeutic decision-making, and clinical outcome of a geriatric dog in which a multidrug-resistant hepatic abscess occurred in association with congenital EHPSS, and to propose a pathophysiologic link between chronic portal hypoperfusion and intrahepatic infection. An 11-year-old neutered male Maltese dog with a known EHPSS presented with acute anorexia and lethargy. Diagnostic imaging revealed a hepatic abscess adjacent to the gallbladder, and cytology confirmed a septic process. Despite targeted meropenem therapy based on antimicrobial susceptibility testing, the abscess failed to regress and C-reactive protein levels continued to rise. Concern for persistent biliary contamination and impaired hepatic immune clearance led to surgical intervention. A combined procedure—partial hepatic lobectomy, cholecystectomy, and shunt attenuation—was performed. Postoperative hypotension was managed successfully with vasopressors and transfusion. The patient recovered uneventfully, and at four-month follow-up, hepatic enzyme activities normalized and liver size increased. These findings highlight the need to evaluate hepatic infections in dogs with EHPSS as a potential consequence of impaired hepatic immune clearance rather than an incidental finding.

## Simple Summary

Hepatic abscesses are rare in dogs and are most commonly associated with biliary disease or ascending bacterial infection. Congenital extrahepatic portosystemic shunts (EHPSS) are known to impair hepatic perfusion and immune clearance; however, their potential role in predisposing dogs to hepatic abscess formation has not been previously reported. This case describes a geriatric dog with congenital EHPSS that developed a multidrug-resistant hepatic abscess unresponsive to targeted antimicrobial therapy. Definitive treatment required a multidisciplinary surgical approach, including partial hepatic lobectomy, cholecystectomy, and shunt attenuation. The clinical course and histopathologic findings suggest that long-standing portosystemic shunting may impair hepatic immune function and contribute to intrahepatic bacterial persistence. This report highlights EHPSS as a potential, previously underrecognized risk factor for hepatic abscess formation and emphasizes the importance of addressing both the infectious focus and the underlying vascular abnormality to achieve successful long-term outcomes in affected dogs.

## 1. Introduction

Hepatic abscesses are uncommon in dogs and are most frequently reported secondary to hepatobiliary disease, abdominal trauma, or ascending bacterial infection from enteric organisms, most notably Escherichia coli [1]. A recent multicenter retrospective study has shown that canine hepatic abscesses are commonly associated with biliary tract disease and carry substantial morbidity, particularly in geriatric patients [1]. Previous reports of hepatic abscesses in dogs have primarily described associations with biliary tract disease, penetrating foreign bodies, abdominal trauma, metabolic disorders such as diabetes mellitus, or postoperative complications following hepatobiliary surgery [2,3,4]. In these studies, hepatic abscesses were most commonly attributed to ascending bacterial infection, with Escherichia coli being the predominant isolate, and treatment strategies ranged from medical management to surgical intervention depending on lesion size, location, and clinical severity [3,5]. More recent reports have emphasized the importance of definitive source control, particularly in cases refractory to antimicrobial therapy or associated with underlying structural disease [5,6]. However, congenital vascular anomalies such as extrahepatic portosystemic shunts have not been previously recognized as a potential predisposing factor for hepatic abscess formation in dogs. Although several risk factors for hepatic abscess formation have been identified, the influence of congenital vascular abnormalities on hepatic infectious susceptibility has not been thoroughly explored. Congenital extrahepatic portosystemic shunts (EHPSS) are a well-recognized vascular anomaly in dogs, resulting in chronic diversion of portal blood flow away from the liver. In canine patients, this leads to hepatic hypoperfusion, hepatocellular atrophy, and impaired hepatic immune surveillance, including reduced Kupffer cell–mediated bacterial clearance [7]. Although direct evidence linking EHPSS to hepatic abscess formation in dogs is lacking, experimental studies in animal models have demonstrated that portocaval shunting impairs hepatic macrophage–mediated bacterial clearance, providing a biologically plausible mechanism for increased susceptibility to intrahepatic infection [8]. To date, an association between congenital EHPSS and hepatic abscess development has not been reported in canine patients, and any potential relationship remains speculative.

Here, we describe a geriatric dog with congenital EHPSS that developed a multidrug-resistant hepatic abscess unresponsive to medical management. This case highlights a possible association between congenital EHPSS and hepatic abscess formation and underscores the importance of evaluating hepatic infections in dogs with portosystemic shunting as a potentially related clinical finding, rather than assuming an incidental coexistence.

## 2. Case Presentation

An 11-year-2-month-old neutered male Maltese dog (3.4 kg) was presented with acute lethargy and anorexia.

Eight months prior to presentation, the dog had undergone abdominal computed tomography as part of the diagnostic evaluation for a generalized tonic–clonic seizure, which confirmed a congenital extrahepatic portosystemic shunt (single, extrahepatic, right gastric–caval type). Retrospective review of the abdominal CT images revealed diffuse hepatic volume reduction with generalized attenuation of intrahepatic portal veins, consistent with portal hypoperfusion. Multiple low-attenuation lesions were identified throughout the liver parenchyma, and the right lateral hepatic lobe showed marked volume loss with multifocal hyperattenuating foci compatible with mineralization. The gallbladder contained multiple hyperattenuating intraluminal materials with mild wall thickening and contrast enhancement, without evidence of intrahepatic bile duct or common bile duct dilation. Importantly, despite the presence of multifocal parenchymal attenuation abnormalities, there was no discrete rim-enhancing lesion, cavitary structure, or localized pericholecystic inflammatory change suggestive of hepatic abscess formation at that time, including in the region where the abscess subsequently developed. The patient was hospitalized for one week and subsequently managed medically with levetiracetam (30 mg/kg PO BID), ursodeoxycholic acid (7.5 mg/kg PO BID), silymarin (7.5 mg/kg PO BID), and lactulose (0.5 mL PO TID), with good clinical response.

On day 1 of hospitalization, laboratory tests revealed an elevated C-reactive protein (CRP) level of 6.1 mg/dL, an inflammatory leukogram, and increased hepatic enzyme activities.

To better characterize hepatic function over time, laboratory parameters reflecting hepatic synthetic and metabolic capacity were reviewed at two clinically relevant time points: (1) at the time of incidental diagnosis of congenital EHPSS during evaluation for seizure activity, and (2) at the time of clinical diagnosis of hepatic abscess. At the time of EHPSS diagnosis, hepatic function indices were relatively preserved, whereas at the time of clinical diagnosis of hepatic abscess, laboratory findings demonstrated progressive hepatic dysfunction accompanied by marked systemic inflammation, including severe leukocytosis and decreased hematocrit (Table S1).

**Figure 1:**
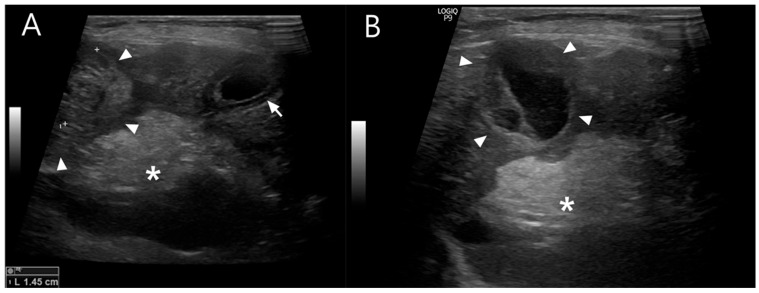
(**A**) Abdominal ultrasonographic image showing the liver and gallbladder. The gallbladder wall is thickened (arrow), displaying a double rim sign, which can be seen in cases of gallbladder edema, hypoalbuminemia, sepsis, or peritonitis. A region of increased echogenicity is present dorsal to the gallbladder (asterisk), suggestive of localized peritonitis. A heterogeneous hepatic nodule is also identified (arrowhead). (**B**) The hepatic nodule (arrowhead) appears round but atypical, containing internal septations and anechoic cyst-like structures. Increased echogenicity of the adjacent peritoneum beneath the nodule (asterisk) is noted, consistent with peritoneal reaction.

**Figure 2:**
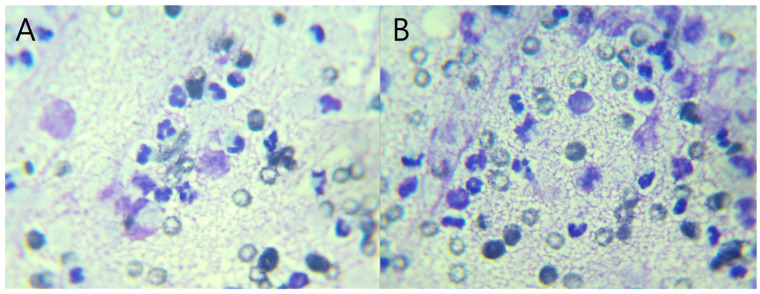
(**A**,**B**) Cytologic images from ultrasound-guided fine-needle aspiration (FNA) of the hepatic nodule. Under oil immersion (×1000 magnification), numerous toxic neutrophils and red blood cells (RBCs) are visible. The background shows a diffuse granular eosinophilic matrix, consistent with necrotic debris.

Abdominal ultrasonography showed progressive gallbladder wall thickening, localized hyperechoic peritonitis, and a heterogeneous hepatic nodule with internal anechoic, cyst-like structures (Figure 1), raising suspicion for hepatic abscess, cholangitis, or hepatitis. Concurrently, ultrasound-guided fine-needle aspiration (FNA) and bacterial culture with antibiotic susceptibility testing were performed. Grossly, the aspirated material was yellowish and slightly turbid, with a viscous, bile-tinged appearance consistent with a septic inflammatory process. Cytologic analysis of the FNA sample revealed numerous toxic neutrophils (Figure 2), and the hepatic nodule was diagnosed as a hepatic abscess.

Potential extrahepatic sources of bacterial seeding were evaluated. No clinical or historical evidence of periodontal disease was identified on oral examination, and urinalysis performed at admission did not support urinary tract infection. Thoracic imaging revealed no findings suggestive of pneumonia or other infectious foci. Blood cultures were not performed, as a focal hepatic lesion was identified and cytologic evaluation confirmed a septic process, allowing targeted antimicrobial therapy based on culture and susceptibility testing of the hepatic aspirate.

Empirical treatment was initiated with triple antibiotic therapy (amoxicillin-clavulanic acid, marbofloxacin, and metronidazole), intravenous fluids, and hepatoprotective agents.

Three days later, culture results identified multidrug-resistant Escherichia coli resistant to the initial antibiotic regimen. Based on the susceptibility profile, antimicrobial therapy was adjusted to meropenem, a carbapenem to which the isolate was susceptible. However, the patient showed no clinical improvement following the change in therapy.

Despite adjustment to meropenem based on antimicrobial susceptibility testing, the hepatic abscess failed to regress. In addition, ultrasonographic findings suggested persistent pericholecystic inflammation, raising concern that the gallbladder might serve as a continuing source of bacterial contamination. Taken together, these findings indicated that medical management alone would be insufficient to resolve the infection, prompting consideration of definitive surgical intervention.

**Figure 3:**
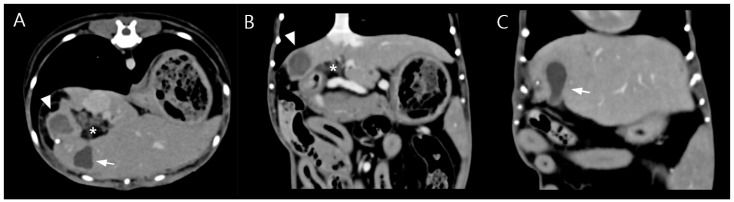
Preoperative contrast-enhanced computed tomography (CT) images (soft tissue window; WL 100, WW 395). (**A**) Transverse image showing a well-defined hypoattenuating lesion adjacent to the gallbladder (arrow) with peripheral rim enhancement (arrowhead), consistent with a hepatic abscess, and surrounding fat stranding (asterisk), suggestive of localized inflammation. (**B**) Dorsal plane reconstruction demonstrating the hepatic abscess at its maximal extent with peripheral rim enhancement (arrowhead). Surrounding fat stranding adjacent to the lesion (asterisk), corresponding to the same region identified in subfigure A, indicates inflammatory changes in the adjacent adipose tissue. (**C**) A more ventral dorsal slice illustrating the gallbladder (arrow), highlighting the close anatomic proximity between the hepatic abscess and the gallbladder–cystic duct junction. Although the hepatic abscess and gallbladder are not visualized within the same dorsal plane slice, their close anatomic relationship is evident across adjacent sections.

Due to poor response to medical management and the potential for disease progression, surgical intervention was elected on day 5 of hospitalization with owner consent. Preoperative CT imaging revealed a hepatic abscess measuring 1.53 × 1.43 × 1.71 cm, located on the caudomedial surface of the right medial hepatic lobe near the junction of the gallbladder and the cystic duct (Figure 3). On contrast-enhanced CT examination, the hepatic abscess and gallbladder were simultaneously visualized on transverse images. On dorsal plane CT reconstructions, the hepatic abscess was most clearly identified on one slice, while the gallbladder was visualized approximately two slices ventral to this level. Although these structures were not visible within the same dorsal plane slice, their close anatomical proximity was evident.

To minimize anesthetic risk in this elderly patient with suspected hepatic insufficiency and incidental cardiac findings, preoperative management included levetiracetam loading (20–40 mg/kg IV), 5% dextrose fluid therapy, and avoidance of midazolam due to its reliance on hepatic metabolism. Pre-anesthetic echocardiography revealed mild concentric left ventricular hypertrophy without chamber dilation or clinical signs of cardiac dysfunction. These findings were considered incidental and of uncertain clinical significance but were factored into anesthetic planning.

General anesthesia was induced and maintained using alfaxalone, isoflurane, and fentanyl—agents selected for minimal hepatic metabolism and cardiovascular impact. Vasopressin constant rate infusion (CRI) and intraoperative blood transfusions were administered to support circulation during hepatic lobectomy. The patient maintained intraoperative systolic blood pressure between 60 and 90 mmHg without the need for inotropic support.

**Figure 4:**
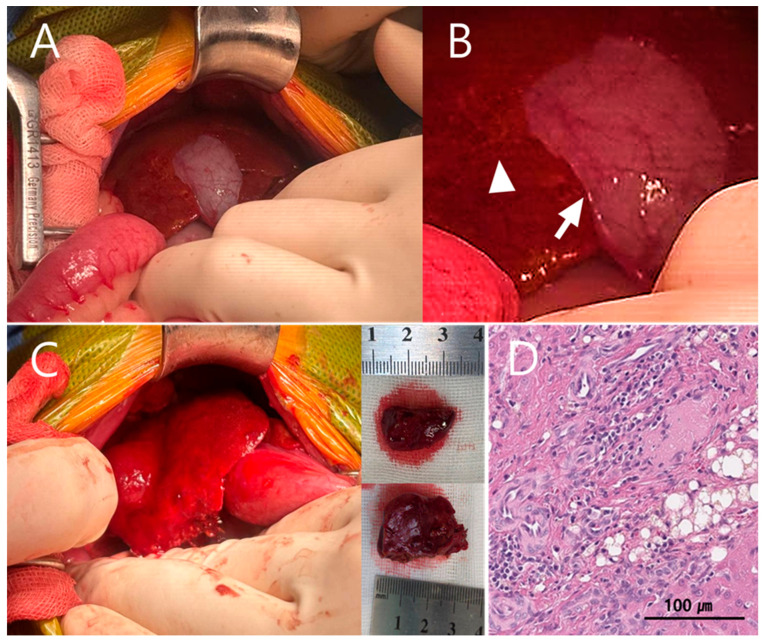
Intraoperative findings following hepatic exposure. (**A**,**B**) Subcapsular edema and pallor are seen on the ventral surface of the right medial lobe. The capsule appears detached, and subcapsular vessels are evident (arrow). A fine, mesh-like yellow pattern (arrowhead) is noted in the adjacent hepatic parenchyma, possibly indicating exudative leakage or edema. (**C**) After cholecystectomy, two abscessed areas were removed via partial hepatic lobectomy using a TA stapler. (**D**) Histopathology revealed a hemorrhagic hepatic abscess with bile duct hyperplasia. Arterial proliferative lesions consistent with microvascular dysplasia (MVD), potentially secondary to portosystemic shunt (PSS), were also identified.

Upon hepatic exposure during surgery, the quadrate lobe and right medial hepatic lobe were identified, with the gallbladder positioned between these lobes (Figure 4A). The hepatic abscess was located deep within the rib cage, and access to the lesion was markedly restricted by the confined subcostal space. Subcapsular edema was noted on the ventral surface of the right medial hepatic lobe, and the hepatic parenchyma appeared yellow and mesh-like (Figure 4A,B). These changes were considered abscess-related, and partial hepatic lobectomy was planned.

Despite careful mobilization, adequate exposure for safe and complete resection of the abscess could not be achieved due to the anatomical position of the gallbladder between the hepatic lobes. Therefore, cholecystectomy was performed as a necessary step to obtain sufficient surgical exposure and achieve definitive source control.

Bile was submitted for bacterial culture at the time of cholecystectomy. The culture results were identical to those obtained from the previous bile aspiration, including the isolated organism and antimicrobial susceptibility profile, supporting the biliary tract as a persistent source of infection.

**Figure 5:**
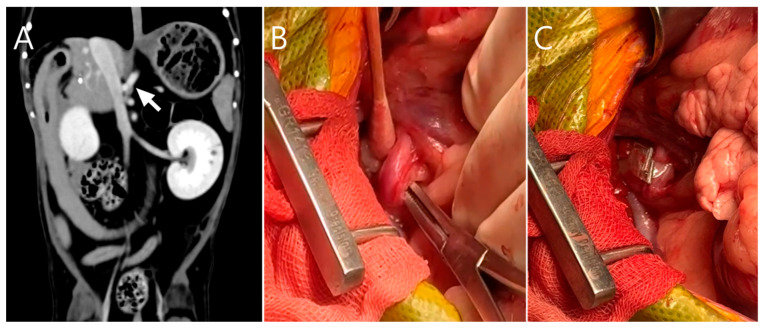
Attenuation of the portosystemic shunt (PSS) post-lobectomy. (**A**) Preoperative CT identifies a shunt originating from the caudal vena cava and coursing to the right gastric vein. The anomalous shunting vessel is indicated by a white arrow. (**B**) Intraoperatively, the shunt was dissected and encircled with a cellophane band (Cellovet^®^). (**C**) The cellophane band was secured with hemoclips.

Following cholecystectomy, two partial hepatic lobectomies were performed using a TA stapler to remove the abscess (Figure 4C). Subsequently, congenital extrahepatic portosystemic shunt attenuation was performed using cellophane banding (Cellovet^®^) (Figure 5). Although intraoperative portal pressure measurement via mesenteric vein catheterization is commonly recommended during shunt attenuation, this technique was intentionally not pursued in the present case because shunt attenuation was performed as part of an extensive combined surgery, and additional vascular manipulation was considered to carry an increased perioperative risk in this septic geriatric patient, given the presence of systemic inflammation, hemodynamic instability, and reduced physiologic reserve.

The most significant hypotensive episode occurred in the immediate postoperative period, with systolic blood pressure declining to 40 mmHg. Over the following 24 h, vasopressin, norepinephrine CRI (0.025–0.05 µg/kg/min), dobutamine CRI (5 µg/kg/min), and repeated transfusions were administered to stabilize hemodynamics. With intensive supportive care, blood pressure gradually normalized.

Histopathological examination of the excised hepatic tissue confirmed a hepatic abscess with bile duct hyperplasia. Additionally, arterial proliferative lesions suggestive of microvascular dysplasia (MVD) were observed (Figure 4D). The proliferative arterial lesions consistent with microvascular dysplasia observed in this patient likely reflect chronic hepatic hypoperfusion, reinforcing the biological plausibility of EHPSS-driven immune dysfunction and impaired bacterial clearance.

Postoperative hypotension was the primary complication and was successfully managed with norepinephrine CRI (0.025–0.05 µg/kg/min), dobutamine CRI (5 µg/kg/min), and transfusions. By 24 h postoperatively, blood pressure was stable without vasopressors and was maintained with intravenous fluids alone. The patient remained hospitalized for continued medical management, including subcutaneous meropenem (10 mg/kg BID). Follow-up ultrasonography revealed no evidence of peritonitis or effusion, and no neurologic abnormalities were observed. By day 14, the CRP level, which had previously remained elevated despite meropenem treatment, had normalized to 1.0, and hepatic enzyme levels returned to pre-illness baseline. The patient’s appetite had fully restored, and outpatient care was resumed.

**Figure 6:**
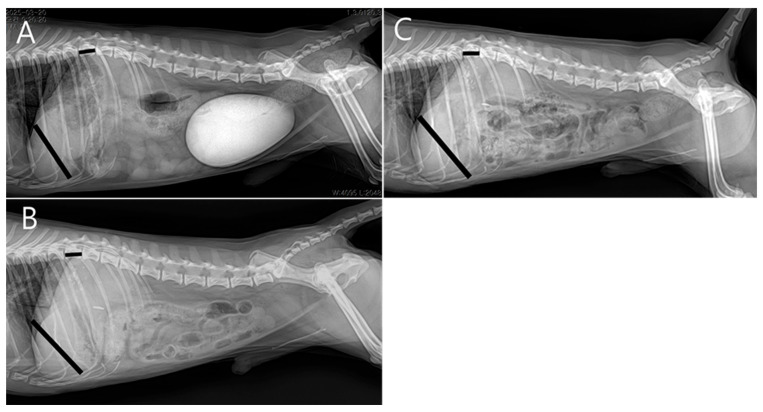
Radiographic liver size evaluation using the liver-to-T11 vertebral length ratio (right lateral view). Measurements were taken from the ventral border of the caudal vena cava to the caudoventral liver apex (liver length), and the midpoint of T11 (vertebral length). Black lines indicate the measured liver length and the T11 vertebral length used to calculate the liver-to-T11 ratio. (**A**) Preoperative ratio: 4.13. (**B**) One month postoperatively: 4.52. (**C**) Four months postoperatively: 4.90, suggesting progressive hepatic enlargement following extrahepatic portosystemic shunt (EHPSS) attenuation.

At the two-week postoperative follow-up, no surgical complications were noted. Subsequent rechecks were spaced at monthly intervals. Four months postoperatively, serum biochemistry showed normal hepatic enzyme levels, and radiographs demonstrated increased liver size compared to preoperative imaging (Figure 6). The patient was receiving levetiracetam (5–10 mg/kg PO BID), ursodeoxycholic acid (10 mg/kg PO BID), and silymarin (10 mg/kg PO BID), with plans for gradual tapering.

## 3. Discussion

Hepatic abscesses are uncommon in dogs and typically occur secondary to hepatobiliary disease, abdominal trauma, or postoperative septic conditions. Ascending infection of enteric bacteria, most notably *Escherichia coli*, through the biliary tract is a well-recognized cause of hepatic abscesses [1].

In the present case, a congenital extrahepatic portosystemic shunt (EHPSS), a type of portosystemic shunt (PSS), was identified as a potential predisposing factor for hepatic abscess formation.

Previous canine hepatic abscess reports have largely focused on biliary disease, penetrating foreign bodies, metabolic disorders, or postoperative infection as predisposing factors [2,3,4,5]. In contrast, the present case suggests a potential association between congenital extrahepatic portosystemic shunt and hepatic abscess formation, representing a previously underrecognized clinical relationship. Unlike prior reports in which hepatic abscesses were attributed to localized biliary pathology or systemic disease, this patient demonstrated long-standing portal hypoperfusion and histopathologic evidence of microvascular dysplasia, consistent with impaired hepatic immune clearance as a biologically plausible mechanism.

However, the hepatic imaging findings observed months before clinical diagnosis—including lobar volume loss and focal mineralization—may not have been solely attributable to chronic hypoperfusion. Such changes could also represent sequelae of prior, clinically unapparent infection or inflammation. In dogs with congenital EHPSS, impaired hepatic immune surveillance may permit subclinical infection to persist or resolve incompletely, leaving residual structural changes detectable on imaging. In retrospect, earlier consideration of blood culture and/or bile culture in EHPSS patients with atypical hepatic imaging findings might have provided additional diagnostic insight and should be considered in similar cases.

This distinction is clinically relevant, as failure to address the underlying portosystemic shunt could predispose affected patients to recurrent intrahepatic infection despite adequate abscess removal and antimicrobial therapy.

Although congenital, clinical signs of hepatic insufficiency did not appear until 10 years of age, suggesting a progressive decline in hepatic function over time. To the authors’ knowledge, this is the first case report to suggest a possible association between congenital EHPSS and hepatic abscess formation, expanding the spectrum of long-term infectious complications potentially associated with chronic hepatic dysfunction.

Portosystemic shunting reduces portal perfusion and contributes to hepatic atrophy, impaired Kupffer cell function, and decreased bacterial clearance [7]. While no prior canine cases have linked PSS to hepatic abscesses, experimental studies in rats have demonstrated that portocaval shunting impairs bacterial clearance by hepatic macrophages [8]. Specifically, Kupffer cell dysfunction following PSS results in reduced elimination of enteric bacteria, thereby increasing the risk of intrahepatic infection [8].

While hepatic abscesses in dogs are typically attributed to biliary tract disease, trauma, or systemic infection, the present case proposes congenital extrahepatic portosystemic shunt as an underrecognized predisposing factor by impairing hepatic immune clearance and facilitating intrahepatic bacterial persistence. This association has not been previously documented in canine patients, suggesting that hepatic infections in dogs with EHPSS may represent a non-incidental association that warrants further investigation.

This finding suggests a plausible dual-mechanism in the present patient: (1) chronic hepatic immune dysfunction associated with long-standing PSS and (2) ascending bacterial infection, evidenced by isolation of multidrug-resistant *Escherichia coli* and ultrasonographic abnormalities in the gallbladder.

The emergence of multidrug-resistant Escherichia coli in this patient highlights the importance of early bacterial culture and antimicrobial susceptibility testing in geriatric dogs with suspected hepatic infections, particularly those with impaired hepatic immune clearance secondary to congenital portosystemic shunting.

In this geriatric patient, the failure of the abscess to respond to targeted antibiotic therapy necessitated surgical intervention. Although less invasive approaches such as percutaneous needle aspiration or catheter drainage are sometimes preferred to preserve hepatic volume, the presence of suspected septic peritonitis on imaging warranted immediate surgical debridement [9]. Given the potential for the gallbladder to act as a persistent source of reinfection, combined with the likelihood of hepatic immune compromise due to PSS, both cholecystectomy and shunt attenuation were deemed necessary. Because persistent portosystemic shunting can perpetuate hepatic immune dysfunction and impair bacterial clearance, failure to correct the EHPSS would have likely predisposed the patient to recurrent intrahepatic infection even after abscess removal. Thus, shunt attenuation in this case was considered not merely adjunctive but an essential component of definitive source control within the specific clinical context of this patient.

Although it is theoretically plausible that correction of congenital EHPSS may contribute to improved long-term infection control by restoring hepatic perfusion and immune clearance, this concept cannot be generalized or immediately justified in the absence of epidemiologic evidence demonstrating an increased prevalence of hepatic abscesses in dogs with EHPSS. In geriatric patients with long-standing shunting, progressive hepatic fibrosis and reduced hepatic reserve may already be present, and shunt attenuation in such cases carries an increased risk of postoperative portal hypertension.

In addition, postattenuation neurologic signs (PANS) remains a well-recognized complication following surgical correction of portosystemic shunts. Therefore, surgical decision-making in elderly dogs with EHPSS should be highly individualized, balancing the potential benefits of improved hepatic perfusion and infection control against the risks of portal hypertension, neurologic complications, and perioperative instability. Thorough preoperative assessment and careful informed consent are essential, with explicit discussion of these potential risks with owners prior to intervention.

Intraoperative portal pressure measurement during shunt attenuation is generally recommended to assess tolerance and the risk of postoperative portal hypertension. However, in the present case, shunt attenuation was performed in conjunction with partial hepatic lobectomy and cholecystectomy in a septic geriatric patient with suspected reduced hepatic reserve. Direct portal pressure measurement via mesenteric vein catheterization was therefore not performed to avoid additional vascular manipulation, prolonged operative time, and increased risk of hemorrhage or hemodynamic instability. This represents a limitation of the present report.

Several limitations of this report should be acknowledged. This is a single isolated case, and therefore its findings cannot be generalized without validation in additional cases or larger studies. Although histopathologic evidence of microvascular dysplasia supports the presence of chronic hepatic hypoperfusion, it does not establish a definitive temporal or causal relationship between congenital EHPSS and hepatic abscess formation. Other unrecognized confounding factors contributing to abscess development cannot be completely excluded. Accordingly, the observations in this report should be interpreted as hypothesis-generating rather than confirmatory.

Additionally, histopathological examination of the gallbladder was not performed in this case; however, bile culture results and the clinical course strongly supported biliary involvement as a persistent source of infection. Nevertheless, intraoperative tolerance to shunt attenuation was assessed based on systemic hemodynamic stability, absence of acute intestinal congestion, and intensive postoperative monitoring, and no clinical evidence of portal hypertension was observed. Furthermore, the anatomical location of the abscess justified cholecystectomy for improved surgical access. Partial hepatic lobectomy was performed using a TA stapler, allowing efficient and safe removal of the abscess with minimal hemorrhage.

While PSS correction in geriatric patients must be carefully weighed—especially in those with comorbidities such as hypertrophic cardiomyopathy (HCM) and minimal hepatic reserve—retrospective studies have demonstrated that dogs over five years of age with surgically corrected congenital EHPSS had significantly longer survival times and fewer clinical signs at 6–12 months post-diagnosis compared to those managed medically. Findings from Beardall et al. [10] support surgical correction even in older patients to improve both longevity and quality of life; in that study, the mean survival time was 24 months in the surgical group versus 8 months in the medically managed group. Nevertheless, surgical decision-making in geriatric cases should remain individualized, taking into account comorbidities, perioperative risks, and the potential for long-term clinical benefit.

Surgical correction in an elderly patient with preexisting cardiac disease and reduced hepatic function presents notable anesthetic and perioperative challenges. In this case, preoperative management included levetiracetam loading, glucose supplementation, and the deliberate avoidance of sedatives with high hepatic metabolism, due to concerns regarding impaired drug clearance and cardiovascular compromise. General anesthesia was induced and maintained with agents selected for minimal hepatic metabolism and cardiovascular impact, including alfaxalone, isoflurane, and fentanyl. During surgery, fentanyl and vasopressin constant rate infusions were administered along with intraoperative blood transfusion to maintain circulation.

Vasopressin was selected over catecholamine inotropes to support vascular tone while minimizing myocardial stimulation, which is particularly important in patients with HCM or sepsis-associated myocardial compromise. In human medicine, vasopressin has been shown to maintain perfusion in septic shock and dynamic left ventricular outflow tract obstruction (LVOTO) without increasing cardiac workload [11].

Despite anticipated hemodynamic instability with hepatic manipulation, the patient maintained systolic blood pressure between 60 and 90 mmHg intraoperatively without requiring inotropic support. However, the most profound hypotension occurred in the immediate postoperative period, when systolic blood pressure declined to 40 mmHg. Over the following 24 h, aggressive hemodynamic support with vasopressin, norepinephrine, dobutamine, and repeated blood transfusions was required, indicating a critical postoperative condition. With intensive supportive care, blood pressure stabilized by 24 h postoperatively.

Although a single etiology could not be definitively established, the clinical course was most consistent with a mixed shock state. Septic shock was considered likely given the presence of a multidrug-resistant hepatic abscess, systemic inflammation, and perioperative manipulation of infected tissue. Hemorrhagic shock may also have contributed, as partial hepatic lobectomy and cholecystectomy inherently carry a risk of blood loss, necessitating repeated transfusions. In addition, vasodilatory effects related to anesthesia and inflammatory mediators may have further exacerbated hypotension.

One possible contributor to the severe hypotensive shock observed immediately after surgery may have been a reduction in venous return secondary to abrupt changes in shunt blood flow following attenuation. Intraoperative portal pressure measurement and hemodynamic monitoring during temporary shunt occlusion are considered important tools for predicting the risk of circulatory compromise during shunt attenuation. In the present case, portal pressure measurement was not performed, representing a limitation of this report. Given the patient’s septic condition and perioperative instability, this decision was made to minimize procedural time and invasiveness; however, portal pressure assessment may have provided valuable information regarding the patient’s hemodynamic tolerance to shunt attenuation. Future cases involving EHPSS correction, particularly in systemically compromised patients, may benefit from routine portal pressure measurement to better inform intraoperative decision-making and risk stratification.

Importantly, early recognition of hemodynamic instability and prompt implementation of a targeted hemodynamic support strategy—including vasopressin to restore vascular tone while minimizing myocardial stress, norepinephrine for pressure support, dobutamine for inotropic support, and transfusion guided by clinical response—were critical to patient stabilization. This case highlights that favorable outcomes following extensive hepatobiliary surgery in septic geriatric patients are achievable only through aggressive but carefully tailored multidisciplinary management rather than avoiding surgical intervention due to perioperative risk. The patient recovered uneventfully and was discharged 7 days after surgery without complications. Serial radiographs showed progressive hepatic enlargement, and follow-up ultrasonography revealed no recurrence of abscess or peritoneal effusion. Four months postoperatively, liver enzyme levels had normalized, and medical therapy was being tapered. The increase in liver size may reflect improved hepatic perfusion and regenerative capacity following PSS attenuation, consistent with prior reports evaluating hepatic volumetric response after shunt ligation [12]. This outcome supports the effectiveness of a multidisciplinary strategy combining abscess removal, cholecystectomy, and shunt attenuation.

This case highlights a potential association between congenital extrahepatic portosystemic shunt and hepatic abscess formation in geriatric dogs, possibly mediated by impaired hepatic immune clearance and intrahepatic bacterial persistence. Early recognition of hepatic abscesses in dogs with EHPSS, combined with prompt antimicrobial susceptibility testing and timely surgical intervention addressing both the infectious focus and the underlying shunt, may prevent septic complications and improve long-term hepatic recovery. These findings underscore the need for clinicians to consider hepatic infection not merely as a coincidental finding in dogs with portosystemic shunting, but as a potentially predictable consequence of chronic hepatic hypoperfusion and immune dysfunction.

## 4. Conclusions

This case report describes the successful multidisciplinary surgical management of a multidrug-resistant hepatic abscess in a geriatric dog with congenital extrahepatic portosystemic shunt after failure of targeted medical therapy. In this geriatric dog, the clinical course and histopathologic findings suggest that long-standing portosystemic shunting may impair hepatic immune clearance and create a permissive environment for intrahepatic bacterial persistence. However, this observation should be interpreted as a first case report suggesting an association, rather than definitive proof of a causal relationship.

In geriatric patients with congenital extrahepatic portosystemic shunt and suspected hepatic infection, early imaging, cytologic evaluation, and bacterial culture with antimicrobial susceptibility testing are essential to guide therapy. When medical management is insufficient and a biliary source of contamination is suspected, timely surgical source control—including abscess removal, cholecystectomy, and shunt attenuation—may be justified even in older dogs, provided that anesthetic and hemodynamic risks are carefully managed. The favorable outcome and evidence of hepatic regeneration observed in the present case suggest that an aggressive but targeted multidisciplinary approach can restore hepatic function and improve long-term prognosis in similar clinical contexts.

## Data Availability

The original contributions presented in this study are included in the article. Further inquiries can be directed to the corresponding author.

## Supplementary Materials

The following supporting information can be downloaded at: https://www.mdpi.com/article/10.3390/vetsci13010037/s1, Table S1: Hepatic function-related laboratory parameters at two time points: (1) at the time of incidental diagnosis of extrahepatic portosystemic shunt (EHPSS) during seizure evaluation; and (2) at the time of clinical diagnosis of hepatic abscess.
