# Supplementary material for: Multidisciplinary Surgical Treatment of Hepatic Abscess in a Geriatric Dog with Congenital Extrahepatic Portosystemic Shunt
Source: Vet Sci. 2026 Jan 1;13(1):37. doi: 10.3390/vetsci13010037 (PMC12846577; doi:10.3390/vetsci13010037)
Supplement: Supplementary file 1 [file vetsci-13-00037-s001.zip › vetsci-4060237-supplementary.pdf]

Table S1. Hepatic function–related laboratory parameters at two time points: (1) at the time of incidental diagnosis of extrahepatic portosystemic shunt (EHPSS) during seizure evaluation, and (2) at the time of clinical diagnosis of hepatic abscess.

Parameter	Reference range	EHPSS incidental diagnosis<br>(2024/08/14)	Hepatic abscess diagnosis<br>(2025/03/21)
**Albumin (g/dL)**	2.2–3.9	2.9	1.5 ↓
**Total protein (g/dL)**	5.2–8.2	6.4	3.9 ↓
**Globulin (g/dL)**	2.5–4.5	3.5	2.5
**Albumin/Globulin ratio**	—	0.8	0.6 ↓
**Total bilirubin (mg/dL)**	0–0.9	<0.1	<0.1
**ALT (U/L)**	10–125	300 ↑	267 ↑
**ALP (U/L)**	23–212	221 ↑	265 ↑
**GGT (U/L)**	0–11	1	*Not available*
**BUN (mg/dL)**	7–27	9	31 ↑
**Creatinine (mg/dL)**	0.5–1.8	0.6	1.4
**Ammonia (µmol/L)**	0–98	154 ↑	*Not available*
**Glucose (mg/dL)**	70–143	97	121
**Cholesterol (mg/dL)**	110–320	135	*Not available*
**CRP (mg/dL)**	0.1–1.0	*Not available*	7.5 ↑
**WBC ($\times 10^9$/L)**	5.05–16.76	9.52	51.76 ↑
**HCT (%)**	37.3–61.7	45.1	30.3 ↓
